# Improved Arabic Alphabet Characters Classification Using Convolutional Neural Networks (CNN)

**DOI:** 10.1155/2022/9965426

**Published:** 2022-01-11

**Authors:** Nesrine Wagaa, Hichem Kallel, Nédra Mellouli

**Affiliations:** ^1^National Institute of Applied Sciences and Technology (INSAT) at University of Carthage, LARATSI Laboratory, Cedex 1080, Tunis, Tunisia; ^2^MedTech at South Mediterranean University, Cedex 1053, Tunis, Tunisia; ^3^Laboratory of Advanced Computer Science at Paris 8 University, LIASD (EA4383), France

## Abstract

Handwritten characters recognition is a challenging research topic. A lot of works have been present to recognize letters of different languages. The availability of Arabic handwritten characters databases is limited. Motivated by this topic of research, we propose a convolution neural network for the classification of Arabic handwritten letters. Also, seven optimization algorithms are performed, and the best algorithm is reported. Faced with few available Arabic handwritten datasets, various data augmentation techniques are implemented to improve the robustness needed for the convolution neural network model. The proposed model is improved by using the dropout regularization method to avoid data overfitting problems. Moreover, suitable change is presented in the choice of optimization algorithms and data augmentation approaches to achieve a good performance. The model has been trained on two Arabic handwritten characters datasets AHCD and Hijja. The proposed algorithm achieved high recognition accuracy of 98.48% and 91.24% on AHCD and Hijja, respectively, outperforming other state-of-the-art models.

## 1. Introduction

Approximately a quarter of a billion people around the world speak and write the Arabic language [1]. There are a lot of historical books and documents that represent a crucial data set for most Arabic countries written in the Arabic language [1, 2].

**Figure 1:**
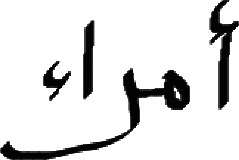
One possible connected component box for the word “أمراء”.

Recently, the area of Arabic handwritten characters recognition (AHCR) has received increased research attention [3–5]. It is a challenging topic of computer vision and pattern recognition [1]. This is due to the following:

- The difference between handwriting patterns [3].
- The form similarity between Arabic alphabets [1, 3].
- The diacritics of Arabic characters [6].
- As shown in Figure 1, in the Arabic language the shape of each handwritten character depends on its position in the world. For example, here in the word “أمراء” the character “Alif” is written in two different forms “أ” and “ا”, where, in the Arabic language, each character has between two and four shapes.  Table 1 shows the different shapes of the twenty-eight Arabic alphabets.

With the development of deep learning (DL), convolution neural networks (CNNs) have shown a significant capability to recognize handwritten characters of different languages [3, 7, 8]: Latin [9, 10], Chine [11], Devanagari [12], Malayalam [11], etc.

Most researchers improved the CNN architecture to achieve good handwritten characters recognition performance [6, 13]. However, a neural network with excellent performance usually requires a good tuning of CNN hyperparameters and a good choice of applied optimization algorithms [14–16]. Also, a large amount of training dataset [17, 18] is required to achieve outstanding performance.

The main contributions of this research can be summarized as follows:

- Suggesting a CNN model for recognizing Arabic handwritten characters.
- Tuning of different hyperparameters to improve the model performance.
- Applying different optimization algorithms. Reporting the effectiveness of the best ones.
- Presenting different data augmentation techniques. Reporting the influence of each method on the improvement of Arabic handwritten characters recognition.
- Mixing two different Arabic handwritten characters datasets for shape varying. Testing the impact of the presented data augmentation approaches on the mixed dataset.

The rest of this paper is organized as follows. In Section 2, we expose the related works in Arabic handwritten character classification. In Sections 3 and 4, we describe the convolution neural network architecture and the model tuning hyperparameters. In Section 5, we make a detailed description of various used optimization algorithms. In Section 6, we describe the different utilized data augmentation techniques chosen in this study. In Section 7, we provide an overview of the experimental results showing the CNN distinguished performance.  Section 8 is conclusion and possible future research directions.

## 2. Related Work

In recent years, many studies have addressed the classification and recognition of letters, including Arabic handwritten characters. On the other hand, there are a smaller number of proposed approaches for recognizing individual characters in the Arabic language. As a result, Arabic handwritten character recognition is less common compared to English, French, Chinese, Devanagari, Hangul, Malayalam, etc.

Impressive results were achieved in the classification of handwritten characters from different languages, using deep learning models and in particular the CNN.

El-Sawy et al. [6] gathered their own Arabic Handwritten Character dataset (AHCD) from 60 participants. AHCD consists of 16.800 characters. They have achieved a classification accuracy of 88% by using a CNN model consisting of 2 convolutional layers. To improve the CNN performance, regularization and different optimization techniques have been implemented to the model. The testing accuracy was improved to 94.93%.

Altwaijry and Turaiki [13] presented a new Arabic handwritten letters dataset (named “Hijja”). It comprised 47.434 characters written by 591 participants. Their proposed CNN model was able to achieve 88% and 97% testing accuracy, using the Hijja and AHCD datasets, respectively.

Younis [19] designed a CNN model to recognize Arabic handwritten characters. The CNN consisted of three convolutional layers followed by one final fully connected layer. The model achieved an accuracy of 94.7% for the AHCD database and 94.8% for the AIA9K (Arabic alphabet's dataset).

Latif et al. [20] designed a CNN to recognize a mix of handwriting of multiple languages: Persian, Devanagari, Eastern Arabic, Urdu, and Western Arabic. The input image is of size (28 × 28) pixels, followed by two convolutional layers, and then a max-pooling operation is applied to both convolution layers. The overall accuracy of the combined multilanguage database was 99.26%. The average accuracy is around 99% for each individual language.

Alrobah and Albahl [21] analyzed the Hijja dataset and found irregularities, such as some distorted letters, blurred symbols, and some blurry characters. They used the CNN model to extract the important features and SVM model for data classification. They achieved a testing accuracy of 96.3%.

Mudhsh et al. [22] designed the VGG net architecture for recognizing Arabic handwritten characters and digits. The model consists of 13 convolutional layers, 2 max-pooling layers, and 3 fully connected layers. Data augmentation and dropout methods were used to avoid the overfitting problem. The model was trained and evaluated by using two different datasets: the ADBase for the Arabic handwritten digits classification topic and HACDB for the Arabic handwritten characters classification task. The model achieved an accuracy of 99.66% and 97.32% for ADBase and HACDB, respectively.

Boufenar et al. [23] used the popular CNN architecture Alexnet. It consists of 5 convolutional layers, 3 max-pooling layers, and 3 fully connected layers. Experiments were conducted on two different databases, OIHACDB-40 and AHCD. Based on the good tuning of the CNN hyperparameters and by using dropout and minibatch techniques, a CNN accuracy of 100% and 99.98% for OIHACDB-40 and AHCD was achieved.

Mustapha et al. [24] proposed a Conditional Deep Convolutional Generative Adversarial Network (CDCGAN) for a guided generation of isolated handwritten Arabic characters. The CDCGAN was trained on the AHCD dataset. They achieved a 10% performance gap between real and generated handwritten Arabic characters.

Table 2 summarizes the literature reviewed for recognizing Arabic handwriting characters using the CNN models. From the previous literature, we notice that most CNN architectures have been trained by using adult Arabic handwriting letters “AHCD”. In addition, we observe that most researchers try to improve the performance through the good tuning of the CNN model hyperparameters.

## 3. The Proposed Arabic Handwritten Characters Recognition System

**Figure 2:**
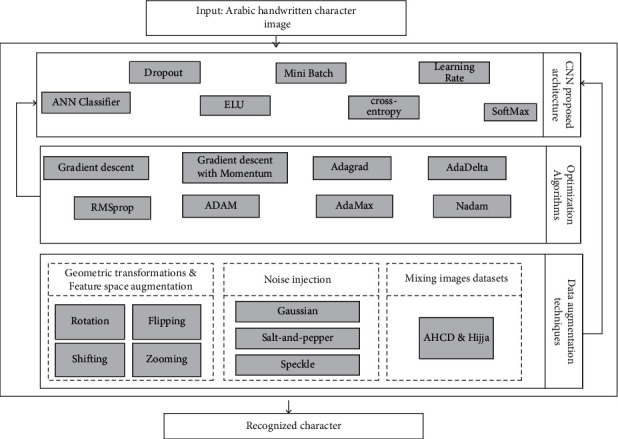
Proposed recognition schema for Arabic handwritten characters datasets.

As shown in Figure 2, the model that we proposed in this study is composed of three principal components: CNN proposed architecture, optimization algorithms, and data augmentation techniques.

In this paper, the proposed CNN model contains four convolution layers, two max-pooling operations, and an ANN model with three fully hidden layers used for the classification. To avoid the overfitting problems and improve the model performance, various optimization techniques were used such as dropout, minipatch, choice of the activation function, etc.

**Figure 3:**
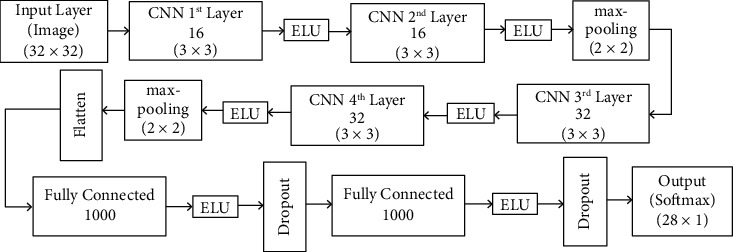
Proposed CNN architecture.

Figure 3 describes the proposed CNN model. Also, in this work, the recognition performance of Arabic handwritten letters was improved through the good choice of the optimization algorithm and by using different data augmentation techniques “geometric transformations, feature space augmentation, noise injection, and mixing images.”

## 4. Convolution Neural Network Architecture

A CNN model [25–34] is a series of convolution layers followed by fully connected layers. Convolution layers allow the extraction of important features from the input data. Fully connected layers are used for the classification of data. The CNN input is the image to be classified; the output corresponds to the predicted class of the Arabic handwritten character.

### 4.1. Input Data

The input data is an image *I* of size (*m* × *m* × *s*). (*m* × *m*) Defines the width and the height of the image and *s* denotes the space or number of channels. The value of *s* is 1 for a grayscale image and equals 3 for a RGB color image.

### 4.2. Convolution Layer

The convolution layer consists of a convolution operation followed by a pooling operation.

#### 4.2.1. Convolution Operation

**Figure 4:**
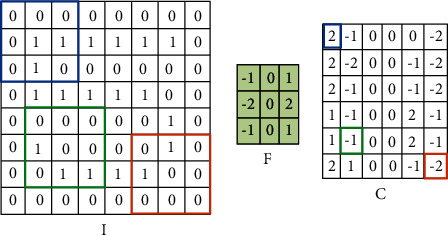
Example of 2D convolution operation [13, 31].

The basic concept of the classical convolution operation between an input image *I* of dimension (*m* × *m*) and a filter *F* of size (*n* × *n*) is defined as follows (see Figure 4):

(1) $$\begin{array}{c} C\;=I\;⊗\;F. \end{array}$$

Here, ⊗ denotes the convolution operation. *C* is the convolution map of size (*a* × *a*), where *a*=(*m* − *n*+2*p*/*sL*)+1. *sL* is the stride and denotes the number of pixels by which *F* *is* sliding  over *I*. *p* is the padding; often it is necessary to add a bounding of zeros around *I* to preserve complete image information. Figure 4 is an example of the convolution operation between an input image of dimension (8 × 8) and a filter *F* of size (3 × 3). Here, the convolution map *C* is of size (6 × 6) with a stride *sL*=1 and a padding *p*=0.

**Figure 5:**
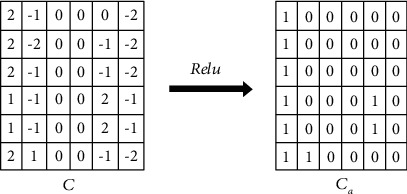
Convolution map after applying ReLU activation function [13, 25, 31].

Generally, a nonlinear activation function is applied on the convolution map C. The commonly used activation functions are Sigmoid [34–36], Hyperbolic Tangent “Tanh” [35, 37], and Rectified Linear Unit “ReLU” [37, 38] where

(2) $$\begin{array}{c} C_{a}=f\left(C\right), \end{array}$$

here, *C*_*a*_ is the convolution map after applying the nonlinear activation function *f.*Figure 5 shows the *C*_*a*_ map when the ReLU activation function is applied on  *C*.

#### 4.2.2. Pooling Operation

**Figure 6:**
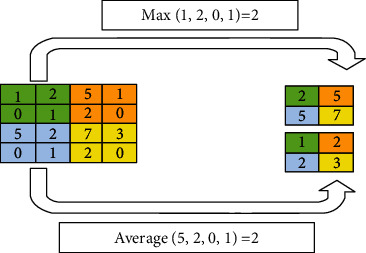
Average-pooling, and max-pooling with filter (2 ×  2) and stride 2 [13, 31].

The pooling operation is used to reduce the dimension of *C*_*a*_ thus reducing the computational complexity of the network. During the pooling operation, a kernel *K* of size (*s*_*p*_ ×  *s*_*p*_) is sliding over  *C*_*a*_. *s*_*p*_ denotes the number of patches by which *K* is sliding over *C*_*a*_. In our analysis *s*_*p*_ is set to 2. The pooling operation is expressed as

(3) $$\begin{array}{c} P=\text{pool}\left(C_{a}\right), \end{array}$$

where *P* is the pooling map and pool is the pooling operation. The commonly used pooling operations are average-pooling, max-pooling, and min-pooling.  Figure 6 describes the concept of average-pooling and max-pooling operations using a kernel of size (2 ×  2) and a stride of 2.

### 4.3. Concatenation Operation

The concatenation operation maps the set of the convoluted images into a vector called the concatenation vector  *Y*.

(4) $$\begin{array}{c} Y=\left[\begin{array}{c} P_{1}^{c}\\ P_{2}^{c}\\ \vdots \\ P_{n}^{c} \end{array}\right], \end{array}$$

here, *P*_*i*_^*c*^ is the output of the *i*^th^ convolution layer. *n* denotes the number of filters applied on the convoluted images *P*_*i*−1_^*c*−1^.

### 4.4. Fully Connected Layer

The CNN classification operation is performed through the fully connected layer [39]. Its input is the concatenation vector *Y*; the predicted class *y* is the output of the CNN classifier. The classification operation is performed through a series of *t* fully connected hidden layers. Each fully connected hidden layer is a parallel collection of artificial neurons. Like synapses in the biological brain, the artificial neurons are connected through weights *W*. The model output of the *i*^th^ fully connected hidden layer is

(5) $$\begin{array}{c} Y^{i}=f\left(H^{i}\right), \end{array}$$

where the weight sum vector *H*^*i*^ is

(6) $$\begin{array}{c} H^{i}=W^{i}Y^{\left(i-1\right)}+\;B^{i}, \end{array}$$

here, *f* is a nonlinear activation function (sigmoid, Tanh, ReLU, etc.). The bias value *B*^*i*^ defines the activation level of the artificial neurons.

## 5. CNN Learning Process

A trained CNN is a system capable of determining the exact class of a given input data. The training is achieved through an update of the layer's parameters (filters, weights, and biases) based on the error between the CNN predicted class and the class label. The CNN learning process is an iterative process based on the feedforward propagation and backpropagation operations.

### 5.1. Feedforward Propagation

For the CNN model, the feedforward equations can be derived from (1)–(5) and (6). The Softmax activation [40, 41] function is applied in the final layer to generate the predicted value of the class of the input image *I*. For a multiclass model, the Softmax is expressed as follows:

(7) $$\begin{array}{c} y_{i}=\frac{\exp\left(h_{i}\right)}{\sum_{j}^{c}\exp\left(h_{j}\right)}, \end{array}$$

where *c* denotes the number of classes,  *y*_*i*_ is the  *i*^th^ coordinate of the output vector *y*, and the artificial neural output *h*_*i*_=∑_*j*=1_^*n*^*h*_*i*_*w*_*ij*_.

### 5.2. Backpropagation

To update the CNN parameters and perform the learning process, a backpropagation optimization algorithm is developed to minimize a selected cost function *E*. In this analysis, the cross-entropy (CE) cost function [40] is used.

(8) $$\begin{array}{c} E=-\sum_{j=1}^{p}\left(\overset{ˇ}{y_{i}}\;\log\left(y_{i}\right)+\left(1+\overset{ˇ}{y_{i}}\right)\log\left(1-y_{i}\right)\right), \end{array}$$

here, $\overset{ˇ}{y_{i}}$ is the desired output (data label).

The most used optimization algorithm to solve classification problems is the gradient descent (GD). Various optimizers for the GD algorithm such as momentum, AdaGrad, RMSprop, Adam, AdaMax, and Nadam were used to improve the CNN performance.

#### 5.2.1. Gradient Descent [40, 42]

GD is the simplest form of optimization gradient descent algorithms. It is easy to implement and gives significant classification accuracy. The general update equation of the CNN parameters using the GD algorithm is

(9) $$\begin{array}{c} \varphi \left(t+1\right)=\;\varphi \left(t\right)-\alpha \frac{\partial E}{\partial \varphi \left(t\right)}, \end{array}$$

where *φ* represents the update of the filters *F*, the weights *W*, and the biases *B*. (∂*E*/∂*φ*) is the gradient with respect to the parameter *φ*.*α* is the model learning rate. A too-large value of *α* may lead to the divergence of the GD algorithm and may cause the oscillation of the model performance. A too-small *α* stops the learning process.

#### 5.2.2. Gradient Descent with Momentum [43]

The momentum hyperparameter *m* defines the velocity by which the learning rate  *α* must be increased when the model approaches to the minimal of the cost function  *E*. The update equations using the momentum GD algorithm are expressed as follows:

(10) $$\begin{array}{c} v\left(t\right)=m\;v\left(t-1\right)+\;\frac{\partial E}{\partial \varphi },\\ \varphi \left(t+1\right)=\varphi \left(t\right)-\;\alpha \;v\left(t\right), \end{array}$$

where *v*(*t*) is the moment gained at *t*^th^ iteration.

#### 5.2.3. AdaGrad [44]

In this algorithm, the learning rate is a function of the gradient (∂*E*/∂*φ*). It is defined as follows:

(11) $$\begin{array}{c} \alpha \left(t\right)=\frac{\beta }{\sqrt{G\left(t\right)+ɛ}}, \end{array}$$

where

(12) $$\begin{array}{c} \beta =\frac{\alpha \left(0\right)}{\sqrt{ɛ}},\\ G\left(t\right)=\sum_{i=1}^{t}{\left(\frac{\partial E}{\partial \varphi \left(t\right)}\right)}^{2}, \end{array}$$

where *ϵ* is a small smoothing value used to avoid the division by 0 and *G*(*t*) is the sum of the squares of the gradients (∂*E*/∂*φ*(*t*)).

With a small magnitude of (∂*E*/∂*φ*), the value of *α* is increasing. If (∂*E*/∂*φ*) is very large, the value of *α* is a constant. AdaGrad optimization algorithm changes the learning rate for each parameter   at a given time *t* with considering the previous gradient update. The parameter update equation using AdaGrad is expressed as follows:

(13) $$\begin{array}{c} \varphi \left(t+1\right)=\varphi \left(t\right)-\;\alpha \left(t\right)\frac{\partial E}{\partial \varphi \left(t\right)}. \end{array}$$

#### 5.2.4. AdaDelta [45]

The issue of AdaGrad is that with much iteration the learning rate becomes very small which leads to a slow convergence. To fix this problem, AdaDelta algorithm proposed to take an exponentially decaying average as a solution, where

(14) $$\begin{array}{c} E\left[G^{2}\left(t\right)\right]=\gamma E\left[G^{2}\left(t-1\right)\right]+\left(1-\gamma \right)G^{2}\left(t\right),\\ \Delta \theta \left(t\right)=\frac{1}{\sqrt{E\left[G^{2}\left(t\right)\right]+\varepsilon }}G\left(t\right),\\ \varphi \left(t+1\right)=\varphi \left(t\right)-\alpha \Delta \theta \left(t\right), \end{array}$$

where *E*[*G*^2^(*t*)] is the decaying average over past squared gradients and *γ* is a set usually around 0.9.

#### 5.2.5. RMSprop [45, 46]

In reality, RMSprop is identical to AdaDelta's initial update vector, which we derived above:

(15) $$\begin{array}{c} E\left[G^{2}\left(t\right)\right]=0.9\;E\left[G^{2}\left(t-1\right)\right]+0.1G^{2}\left(t\right),\\ \varphi \left(t+1\right)=\varphi \left(t\right)-\alpha \Delta \theta \left(t\right). \end{array}$$

#### 5.2.6. ADAM [17, 45, 46]

This gradient descent optimizer algorithm computes the learning rate *α* based on two vectors:

(16) $$\begin{array}{c} r\left(t\right)=\beta_{1}r\left(t-1\right)+\left(1-\beta_{1}\right)\frac{\partial E}{\partial \varphi \left(t\right)},\\ v\left(t\right)=\beta_{2}v\left(t-1\right)+\left(1-\beta_{2}\right){\left(\frac{\partial E}{\partial \varphi \left(t\right)}\right)}^{2}, \end{array}$$

where *r*(*t*) and *v*(*t*) are the 1^st^ and the 2^nd^ order moments vectors. *β*_1_ and *β*_2_ are the decay rates. *r*(*t* − 1) and *v*(*t* − 1) represent the mean and the variance of the previous gradient.

When *r*(*t*) and *v*(*t*) are very small, a large step size is needed for parameters update. To avoid this issue, a bias correction value is added to *r*(*t*) and *v*(*t*).

(17) $$\begin{array}{c} \hat{r}\left(t\right)=\;\frac{r\left(t\right)}{\left(1-\beta_{1}^{t}\right)},\\ \hat{v}\left(t\right)=\;\frac{v\left(t\right)}{\left(1-\beta_{2}^{t}\right)}, \end{array}$$

where *β*_1_^*t*^ is *β*_1_ power *t* and *β*_2_^*t*^ is *β*_2_ power *t*.

The Adam update equation is expressed as follows:

(18) $$\begin{array}{c} \varphi \left(t+1\right)=\varphi \left(t\right)-\;\alpha \frac{\hat{r}\left(t\right)}{\sqrt{\hat{v}\left(t\right)+\;ɛ}}. \end{array}$$

#### 5.2.7. AdaMax [45, 47]

The factor *v*(*t*) in the Adam algorithm adjusts the gradient inversely proportionate to the *ℓ*2 norm of previous gradients (via the *v*(*t* − 1)) and current gradient *t*(∂*E*/∂*φ*(*t*)) :

(19) $$\begin{array}{c} v\left(t\right)=\beta_{2}v\left(t-1\right)+\left(1-\beta_{2}\right){\left|\frac{\partial E}{\partial \varphi \left(t\right)}\right|}^{2}. \end{array}$$

The generalization of this update to the *ℓp* norm is as follows:

(20) $$\begin{array}{c} v\left(t\right)=\beta_{2}^{p}v\left(t-1\right)+\left(1-\beta_{2}^{p}\right){\left|\frac{\partial E}{\partial \varphi \left(t\right)}\right|}^{p}. \end{array}$$

To avoid being numerically unstable, ℓ1 and ℓ2 norms are most common in practice. However, in general ℓ∞ also shows stable behavior. As a result, the authors propose AdaMax and demonstrate that *v*(*t*) with ℓ∞ converges to the more stable value. Here,

(21) $$\begin{array}{c} u\left(t\right)=\beta_{2}^{\infty }v\left(t-1\right)+\left(1-\beta_{2}^{\infty }\right){\left|\frac{\partial E}{\partial \varphi \left(t\right)}\right|}^{\infty }=\max\left(\beta_{2}.\;v\left(t-1\right),\;\left|\frac{\partial E}{\partial \varphi \left(t\right)}\right|\;\right),\\ \varphi \left(t+1\right)=\varphi \left(t\right)-\;\alpha \frac{\hat{r}\left(t\right)}{u\left(t\right)}. \end{array}$$

#### 5.2.8. Nadam [43]

It is a combination of Adam and NAG, where the parameters update equation using NAG is defined as follows:

(22) $$\begin{array}{c} v\left(t\right)=\gamma \;v\left(t-1\right)+\alpha \left(\frac{\partial E}{\partial \varphi \left(t\right)}-\gamma \;v\left(t-1\right)\right),\\ \varphi \left(t+1\right)=\varphi \left(t\right)-\;\alpha v\left(t\right). \end{array}$$

The update equation using Nadam is expressed as follows:

(23) $$\begin{array}{c} r=\left(\beta_{1}\hat{r}\left(t\right)+\;\frac{\left(1-\beta_{1}\right)}{1-\beta {\left(t\right)}_{1}}\;\frac{\partial E}{\partial \varphi \left(t\right)}\right),\\ \varphi \left(t+1\right)=\varphi \left(t\right)-\frac{\alpha }{\sqrt{\hat{v}\left(t\right)+ɛ}}r. \end{array}$$

## 6. Data Augmentation Techniques

Deep convolutional neural networks are heavily reliant on big data to achieve excellent performance and avoid the overfitting problem.

To solve the problem of insufficient data for Arabic handwritten characters, we present some basic data augmentation techniques that enhance the size and quality of training datasets.

The image augmentation approaches used in this study include geometric transformations, feature space augmentation, noise injection, and mixing images.

Data augmentation based on geometric transformations and feature space augmentation [17, 48] is often related to the application of rotation, flipping, shifting, and zooming.

### 6.1. Rotation

The input data is rotated right or left on an axis between 1° and 359°. The rotation degree parameter has a significant impact on the safety of the dataset. For example, on digit identification tasks like MNIST, slight rotations like 1 to 20 or −1 to −20 could be useful, but when the rotation degree increases, properly the CNN network cannot accurately distinguish between some digits.

### 6.2. Flipping

The input image is flipped horizontally or vertically. This augmentation is one of the simplest to implement and has proven useful on some datasets such as ImageNet and CIFAR-10.

### 6.3. Shifting

**Figure 7:**
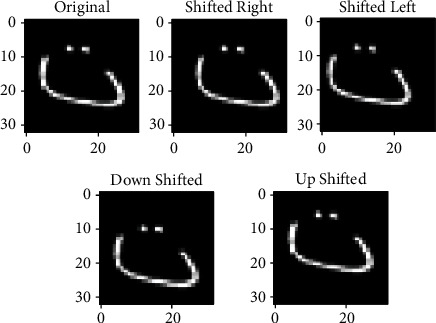
Data augmentation through shifting a single data in four directions by two pixels.

The input image is shifting right, left, up, or down. This transformation is a highly effective adjustment to prevent positional bias.  Figure 7 shows an example of shifting data augmentation technique using Arabic alphabet characters.

### 6.4. Zooming

The input image is zooming, either by adding some pixels around the image or by applying random zooms to the image. The amount of zooming has an influence on the quality of the image; for example, if we apply a lot of zooming, we can lose some image pixels.

### 6.5. Noise Injection

As it could be seen on Arabic handwritten characters, natural noises are presented in images. Noises make recognition more difficult and for this reason, noises are reduced by image preprocessing techniques. The cos of noise reduction is to perform a high classification, but it causes the alteration of the character shape. The main datasets in this research topic are considered with denoising images. The question which we answer here is how the method could be robust to any noise.

Adding noise [48, 49] to a convolution neural network during training helps the model learn more robust features, resulting in better performance and faster learning. We can add several types of noise when recognizing images, such as the following.

- Gaussian noise: injecting a matrix of random values drawn from a Gaussian distribution
- Salt-and-pepper noise: changing randomly a certain amount of the pixels to completely white or completely black
- Speckle noise: only adding black pixels “pepper” or white pixels “salt”

Adding noise to the input data is the most commonly used approach, but during training, we can add random noise to other parts of the CNN model. Some examples include the following:

- Adding noise to the outputs of each layer
- Adding noise to the gradients to update the model parameters
- Adding noise to the target variables

### 6.6. Mixing Image's Databases

In this study, we augment the training dataset by mixing two different Arabic handwritten characters datasets, AHCD and Hijja, respectively. AHCD is a clean database, but Hijja is a dataset with very low-resolution images. It comprises many distorted alphabets images.

Then, we evaluate the influence of different mentioned data augmentation techniques (geometric transformations, feature space augmentation, and noise injection) on the recognition performance of the new mixing dataset.

## 7. Experimental Results and Discussion

### 7.1. Datasets

In this study, two datasets of Arabic handwritten characters were used: Arabic handwritten characters dataset “AHCD” and Hijja dataset.

AHCD [6] comprises 16.800 handwritten characters of size (32 × 32 × 1) pixels. It was written by 60 participants between the ages of 19 and 40 years and most of the participants are right handed. Each participant wrote the Arabic alphabet from “alef” to “yeh” 10 times. The dataset has 28 classes. It is divided into a training set of 13.440 characters and a testing set of 3.360 characters.

Hijja dataset [13] consists of 4.434 Arabic characters of size (32 × 32 × 1) pixels. It was written by 591 school children ranging in age between 7 to 12 years. Collecting data from children is a very hard task. Malformed characters are characteristic of children's handwriting; therefore the dataset comprises repeated letters, missing letters, and many distorted or unclear characters. The dataset has 29 classes. It is divided into a training set of 37.933 characters and a testing set of 9.501 characters (80% for training and 20% for test).

**Figure 8:**
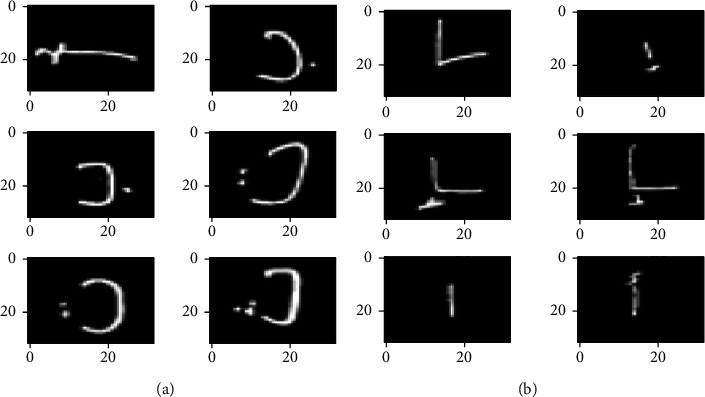
Arabic handwritten letters dataset. (a) AHCD dataset. (b) Hijja dataset.

Figure 8 shows a sample of AHCD and Hijja Arabic handwritten letters datasets.

### 7.2. Experimental Environment and Performance Evaluation

In this study the implementation and the evaluation of the CNN model are done out in Keras deep learning environment with TensorFlow backend on Google Colab using GPU accelerator.

We evaluate the performance of our proposed model via the following measures:

Accuracy (*A*) is a measure for how many correct predictions your model made for the complete test dataset:
  (24) $$\begin{array}{c} A=\frac{\text{TP}+\text{TN}}{\text{TP}+\text{TN}+\text{FN}+\text{FP}}. \end{array}$$
Recall (*R*) is the fraction of images that are correctly classified over the total number of images that belong to class:
  (25) $$\begin{array}{c} R=\frac{\text{TP}}{\text{TP}+\text{FN}}. \end{array}$$
Precision (*P*) is the fraction of images that are correctly classified over the total number of images classified:
  (26) $$\begin{array}{c} P=\frac{\text{TP}}{\text{TP}+\text{FP}}. \end{array}$$
*F*1 measure is a combination of Recall and Precision measures:
  (27) $$\begin{array}{c} F1=2*\frac{P*R}{P+R}. \end{array}$$

Here, TP = true positive (is the total number of images that can be correctly labeled as belonging to a class x), FP = false positive (represents the total number of images that have been incorrectly labeled as belonging to a class x), FN = false negative (represents the total number of images that have been incorrectly labeled as not belonging to a class x), *TN* = true negative (represents the total number of images that have been correctly labeled as not belonging to a class x).

Also we draw the area under the ROC curve (AUC), where we have the following.

An ROC curve (receiver operating characteristic curve) is a graph showing the performance of all classification thresholds. This curve plots two parameters:

- True-positive rate
- False-positive rate

AUC stands for “area under the ROC curve.” That is, AUC measures the entire two-dimensional area underneath the entire ROC curve from (0.0) to (1.1).

### 7.3. Tuning of CNN Hyperparameters

The objective is to choose the best model that fits the AHCD and Hijja datasets well. Many try-and-error trials in the network configuration tuning mechanism were performed.

The best performance was achieved when the CNN model was constructed of four convolution layers followed by three fully connected hidden layers. The model starts with two convolution layers with 16 filters of size (3 × 3), then the remaining 2 convolution layers are with 32 filters of size (3 × 3), and each two convolution layers are followed by max-pooling layers with (2 × 2) kernel dimension. Finally, three fully connected layers (dense layers) with Softmax activation function to perform prediction. ELU, a nonlinear activation function, was used to remove negative values by converting them into 0.001. The values of weights and bias are updated by a backward propagation process to minimize the loss function.

To reduce the overfitting problem a dropout of 0.6 rate is added to a model between the dense layers and applies to outputs of the prior layer that are fed to the subsequent layer. The optimized parameters used to improve the CNN performance were as follows: Optimizer algorithm is Adam, the loss function is the cross-entropy, learning rate = 0.001, batch size = 16, and epochs = 40.

We compare our model to CNN-for-AHCD over both the Hijja dataset and the AHCD dataset. The code for CNN-for-AHCD is available online [31], which allows comparison of its performance over various datasets.

On the Hijja dataset, which has 29 classes, our model achieved an average overall test set accuracy of 88.46%, precision of 87.98%, recall of 88.46%, and an F1 score of 88.47%, while CNN-for-AHCD achieved an average overall test set accuracy of 80%, precision of 80.79%, recall of 80.47%, and an F1 score of 80.4%.

On the AHCD dataset, which has 28 classes, our model achieved an average overall test set accuracy of 96.66%, precision of 96.75%, recall of 96.67%, and an F1 score of 96.67%, while CNN-for-AHCD achieved an average overall test set accuracy of 93.84%, precision of 93.99%, recall of 93.84%, and an F1 score of 93.84%.

The detailed metrics are reported per character in Table 3.

We note that our model outperforms CNN-for-AHCD by a large margin on all metrics.

**Figure 9:**
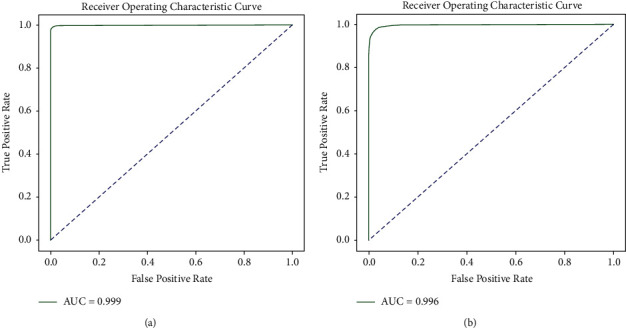
AUC curve of Arabic handwritten characters dataset. (a) AUC of AHCD testing dataset. (b) AUC of Hijja testing dataset.

Figure 9 shows the testing result AUC of AHCD and Hijja dataset.

### 7.4. Optimizer Algorithms

The objective is to choose the best optimizers algorithms that fit the AHCD and Hijja best performance. In this context, we tested the influence of the following algorithms on the classification of handwritten Arabic characters:

- Adam
- SGD
- RMSprop
- AdaGrad
- Nadam
- Momentum
- AdaMax

By using Nadam optimization algorithm, on the Hijja dataset, our model achieved an average overall test set accuracy of 88.57%, precision of 87.86%, recall of 87.98%, and an F1 score of 87.95%.

On the AHCD dataset, our model achieved an average overall test set accuracy of 96.73%, precision of 96.80%, recall of 96.73%, and an F1 score of 96.72%.

The detailed results of different optimizations algorithms are mentioned in Table 4.

### 7.5. Results of Data Augmentation Techniques

Generally, the neural network performance is improved through the good tuning of the model hyperparameters. Such improvement in the CNN accuracy is linked to the availability of training dataset. However, the networks are heavily reliant on big data to avoid overfitting problem and perform well.

Data augmentation is the solution to the problem of limited data. The image augmentation techniques used and discussed in this study include geometric transformations and feature space augmentation (rotation, shifting, flipping, and zooming), noise injection, and mixing images from two different datasets.

**Figure 10:**
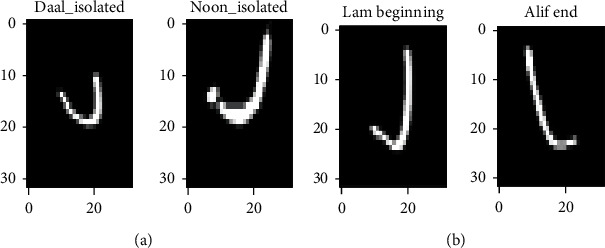
Example of Arabic handwritten characters that cannot be properly distinguished when applying the rotation and flipping data augmentation techniques. (a) Confusion caused by rotation. (b) Confusion caused by flipping.

For the geometric transformations and feature space augmentation, we try to well choose the percentage of rotation, shifting, flipping, and zooming for the model attending a good performance. For example, if we rotate the Latin handwritten number database (MNIST) by 180°, the network will not be able to accurately distinguish between the handwritten digits “6” and “9”. Likewise, on the AHCD and Hijja datasets, if rotating or flipping techniques are used the network will be unable to distinguish between some handwritten Arabic characters. For example, as shown in Figure 10, with a rotation of 180°, the character Daal isolated (د) will be the same as the character Noon isolated (ن).

The detailed results of rotation, shifting, flipping, and zooming data augmentation techniques are mentioned in Table 5.

**Figure 11:**
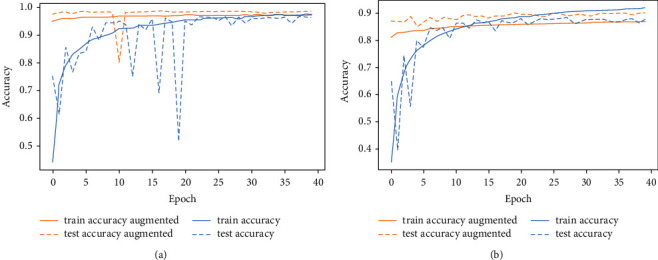
Rotation and shifting data augmentation results of Arabic handwritten characters datasets. (a) Rotation and shifting results of AHCD dataset. (b) Rotation and shifting results of Hijja dataset.

As shown in Table 5 and Figure 11, by using rotation and shifting augmentation approaches, our model achieved a testing accuracy of 98.48% and 91.24% on AHCD dataset and Hijja dataset, respectively. We achieved this accuracy through rotating the input image by 10° and shifting it just by one pixel.

Adding noise is a technique used to augment the training input data. Also in most of the cases, this is bound to increase the robustness of our network.

In this work we used the three types of noise to augment our data:

- Gaussian noise
- Salt-and-pepper noise
- Speckle noise

The detailed results of different types of noise injection are mentioned in Table 6. As shown by adding different types of noise, the model accuracy is improved, which demonstrate the robustness of our proposed architecture. We achieved good results when adding noise to the outputs of each layer.

The proposed idea in this study is to augment the number of training databases by mixing the two datasets AHCD and Hijja, and then we apply the previously mentioned data augmentation methods on the new mixed dataset. Our purpose to use malformed handwritten characters as it proposes the Hijja dataset is to improve the accuracy of our method with noised data.

The detailed results of data augmentation techniques on the mixed database are mentioned in Table 7. As shown, the model performance depends on the rate of using Arabic handwriting “Hijja” database. The children had trouble following the reference paper, which results in very low-resolution images comprising many unclear characters. Therefore mixing the datasets would certainly reduce performance.

## 8. Conclusions and Possible Future Research Directions

In this paper, we proposed a convolution neural network (CNN) to recognize Arabic handwritten characters dataset. We have trained the model on two Arabic datasets AHCD and Hijja. By the good tuning of the network hyperparameters, we achieved an accuracy of 96.73% and 88.57% on AHCD and Hijja.

To improve the model performance, we have implemented different optimization algorithms. For both databases, we achieved an excellent performance by using Nadam optimizer.

To solve the problem of insufficient Arabic handwritten datasets, we have applied different data augmentation techniques. The augmentation approaches are based on geometric transformation, feature space augmentation, noise injection, and mixing of datasets.

By using rotation and shifting techniques, we achieved a good accuracy equal to 98.48% and 91.24% on AHCD and Hijja.

To improve the robustness of the CNN model and increase the number of training datasets, we added three types of noise (Gaussian noise, Salt-and-pepper, and Speckle noise).

Also in this work we first augmented the database by mixing two Arabic handwritten characters datasets; then we tested the results of the previously mentioned data augmentation techniques on the new mixed dataset, where the first database “AHCD” comprises clear images with a very good resolution, but the second database “Hijja” has many distorted characters. Experimentally show that the geometric transformations (rotation, shifting, and flipping), feature space augmentation, and noise injection always improve the network performance, but the rate of using the unclean database “Hijja” harms the model accuracy.

An interesting future direction is the cleaning and processing of Hijja dataset to eliminate the problem of low-resolution and unclear images and then the implementation of the proposed CNN network and data augmentation techniques on the new mixed and cleaned database.

In addition, we are interested in evaluating the result of other augmentation approaches, like adversarial training, neural style transfer, and generative adversarial networks on the recognition of Arabic handwritten characters dataset. We plan to incorporate our work into an application for children that teaches Arabic spelling.

## Figures and Tables

**Table 1:** Twenty-eight shapes of Arabic alphabets.

No.	Name	Isolated	Beginning	Middle	End
1	Alif	ا	ا	ـإ	ـأ
2	Baa	ب	بـ	ـبـ	ـب
3	Tea	ت	تـ	ـتـ	ـت
4	Thea	ث	ثـ	ـثـ	ـث
5	Jam	ج	جـ	ـجـ	ـج
6	Haa	ح	حـ	ـحـ	ـح
7	Khaa	خ	خـ	ـخـ	ـخ
8	Daal	د	د	ـد	ـد
9	Thaal	ذ	ذ	ـذ	ـذ
10	Raa	ر	ر	ـر	ـر
11	Zaay	ز	ز	ـز	ـز
12	Seen	س	سـ	ـسـ	ـس
13	Sheen	ش	شـ	ـشـ	ـش
14	Sad	ص	صـ	ـصـ	ـص
15	Dhad	ض	ضـ	ـضـ	ـض
16	Tah	ط	طـ	ـطـ	ـط
17	Dha	ظ	ظـ	ـظـ	ـظ
18	Ain	ع	عـ	ـعـ	ـع
19	Ghen	غ	غـ	ـغـ	ـغ
20	Fa	ف	فـ	ـفـ	ـف
21	Qaf	ق	قـ	ـقـ	ـق
22	Kaf	ك	كـ	ـكـ	ـك
23	Lam	ل	لـ	ـلـ	ـل
24	Meem	م	مـ	ـمـ	ـم
25	Noon	ن	نـ	ـنـ	ـن
26	Ha	ه	هـ	ـهـ	ـه
27	Waw	و	و	ـو	ـو
28	Yaa	ي	يـ	ـيـ	ـي

**Table 2:** Summary of Arabic handwritten characters recognition using CNN model.

References	Year	Dataset	Type (size)	Method	Optimization	Accuracy (%)	Loss (%)
El-Sawy et al. [6]	2017	AHCD	Chars (16,800)	CNN	(i) Minibatch	94.93	5.1

Mudhsh et al. [22]	2017	ADBase	Digits (6.600)	CNN (based on VGG net)	(ii) Dropout	99.6	—
		HACDB	Chars (70.000)		(iii) Data augmentation	97.32	—

Boufenar et al. [23]	2017	OIHACDB	Chars (6.600)	CNN (based on Alexnet)	(i) Dropout	100	—
		AHCD			(ii) Minibatch	99.98	

Younis [19]	2018	AHCD	Chars (8.737)	CNN	—	97.7	—
		AIA9K				94.8	—

Latif et al. [20]	2018	Mix of handwriting of multiple languages	Chars	CNN	—	99.26	0.02

Altwaijry and Turaiki [13]	2020	Hijja	Chars (47,434)	CNN	—	88	—
		AHCD				97	—

Alrobah &Albahl [21]	2021	Hijja	Chars (47,434)	CNN + SVM	—	96.3	—

Mustapha et al. [24]	2021	AHCD		CDCGAN	—	—	—

**Table 3:** Experimental results on the Hijja and AHCD datasets via CNN-for-AHCD model.

Characters	Hijja dataset						AHCD dataset					
	CNN-for-AHCD			Our model			CNN-for-AHCD			Our model		
	P	R	F1	P	R	F1	P	R	F1	P	R	F1
1. Alif	0.93	0.97	0.95	0.97	0.98	0.98	0.96	0.99	0.98	0.96	1.00	0.98
2. Baa	0.82	0.91	0.86	0.93	0.96	0.94	0.97	0.97	0.97	0.99	0.97	0.98
3. Tea	0.66	0.88	0.75	0.84	0.92	0.88	0.87	0.95	0.91	0.96	0.95	0.95
4. Thea	0.76	0.81	0.78	0.94	0.93	0.93	0.95	0.88	0.92	0.97	0.95	0.96
5. Jam	0.79	0.85	0.82	0.80	0.90	0.84	0.95	0.96	0.95	0.99	0.98	0.99
6. Haa	0.83	0.60	0.70	0.85	0.89	0.87	0.93	0.93	0.93	0.99	0.96	0.97
7. Khaa	0.76	0.77	0.77	0.82	0.73	0.77	0.94	0.93	0.93	0.99	0.96	0.97
8. Daal	0.65	0.69	0.67	0.79	0.68	0.73	0.91	0.94	0.93	0.95	0.88	0.92
9. Thaal	0.70	0.68	0.69	0.83	0.92	0.87	0.96	0.91	0.93	0.88	0.95	0.92
10. Raa	0.86	0.87	0.87	0.81	0.91	0.86	0.89	0.98	0.94	0.94	0.99	0.96
11. Zaay	0.87	0.89	0.88	0.96	0.90	0.93	0.94	0.88	0.91	0.96	0.92	0.94
12. Seen	0.84	0.92	0.88	0.95	0.92	0.94	0.95	0.91	0.93	1.00	0.97	0.99
13. Sheen	0.86	0.82	0.84	0.90	0.88	0.89	0.92	0.98	0.95	0.99	1.00	1.00
14. Sad	0.75	0.81	0.78	0.90	0.86	0.88	0.84	0.96	0.90	0.96	0.97	0.97
15. Dhad	0.80	0.76	0.78	0.91	0.93	0.92	1.00	0.89	0.94	0.97	0.95	0.96
16. Tah	0.90	0.83	0.87	0.96	0.89	0.92	0.96	0.94	0.95	0.94	0.98	0.96
17. Dha	0.83	0.87	0.85	0.84	0.82	0.83	0.97	0.94	0.95	0.97	0.95	0.96
18. Ain	0.74	0.70	0.71	0.86	0.86	0.86	0.95	0.90	0.92	0.98	0.98	0.98
19. Ghen	0.83	0.71	0.77	0.75	0.86	0.80	0.89	0.97	0.93	0.98	0.99	0.99
20. Fa	0.77	0.65	0.71	0.92	0.87	0.89	0.92	0.84	0.88	0.94	0.98	0.96
21. Qaf	0.81	0.80	0.81	0.87	0.89	0.88	0.87	0.91	0.89	0.99	0.95	0.97
22. Kaf	0.86	0.78	0.82	0.93	0.89	0.91	0.98	0.96	0.97	0.98	0.93	0.96
23. Lam	0.90	0.87	0.88	0.91	0.89	0.90	0.98	0.97	0.98	0.99	0.98	0.99
24. Meem	0.83	0.85	0.84	0.82	0.80	0.81	0.98	0.98	0.98	0.98	0.98	0.98
25. Noon	0.70	0.77	0.73	0.88	0.83	0.86	0.92	0.92	0.92	0.86	0.97	0.91
26. Ha	0.81	0.76	0.78	0.89	0.94	0.91	0.97	0.96	0.96	0.97	0.96	0.97
27. Waw	0.930	0.82	0.87	0.94	0.92	0.93	0.96	0.94	0.95	0.97	0.94	0.95
28. Yaa	0.82	0.81	0.82	0.87	0.82	0.84	0.97	0.97	0.97	0.99	0.98	0.99
29. Hamza	0.74	0.73	0.74				na	na	na	na	na	na
Acc. (train)			0.88			**0.98**			0.91			**1.00**
Acc. (test)			0.80			**0.88**			0.94			**0.96**
Macro avg	0.81	0.80	0.80	0.88	0.88	0.88	0.94	0.94	0.94	0.97	0.96	0.96
Weighted avg	0.81	0.80	0.80	0.89	0.88	0.88	0.94	0.94	0.94	0.97	0.96	0.96

**Table 4:** Experimental results on the Hijja and AHCD through different optimizers algorithms.

Algorithm	Accuracy (%)				Precision (%)				Recall (%)				F1 score (%)			
	AHCD		Hijja		AHCD		Hijja		AHCD		Hijja		AHCD		Hijja	
	Training	Testing	Training	Testing	Training	Testing	Training	Testing	Training	Testing	Training	Testing	Training	Testing	Training	Testing
Adam	99.76	96.66	97.60	88.46	99.76	96.75	97.40	87.98	99.76	96.67	97.60	88.46	99.76	96.67	97.60	88.47
SGD	99.44	95.68	94.16	87.85	99.45	95.84	94.28	87.93	99.45	95.68	94.16	87.85	99.45	95.70	94.17	87.88
RMSprop	99.72	96.22	96.55	87.98	99.73	96.30	94.28	87.86	99.72	96.22	94.16	87.98	99.72	96.23	94.17	87.95
AdaGrad	88.62	83.54	63.90	60.37	88.73	83.78	87.86	64.95	88.62	83.54	87.98	63.91	88.60	83.52	87.95	63.77
Nadam	**99.77**	**96.73**	**97.76**	**88.57**	**99.77**	**96.80**	**97.57**	**87.86**	**99.77**	**96.73**	**97.76**	**87.98**	**99.77**	**96.72**	**97.76**	**87.95**
Momentum	99.16	95.56	94.16	87.76	99.18	95.72	94.11	87.73	99.17	95.57	94.17	87.77	99.17	95.58	94.15	87.74
AdaMax	99.89	96.19	97.19	87.57	99.90	96.25	97.01	87.86	99.90	96.19	97.19	87.98	99.90	96.19	97.20	87.95

**Table 5:** Experimental results of data augmentation techniques on Hijja and AHCD datasets.

Dataset	Data augmentation techniques with using Nadam algorithm									
	Rotation		Shifting		Rotation and shifting		Flipping		Zooming	
	Training accuracy	Testing accuracy	Training accuracy	Testing accuracy	Training accuracy	Testing accuracy	Training accuracy	Testing accuracy	Training accuracy	Testing accuracy
AHCD	99.82	97.64	99.56	98.09	99.41	**98.48**	99.69	97.29	99.85	98.00
Hijja	97.18	89.86	92.72	90.28	91.73	**91.24**	95.39	88.94	99.16	88.66

**Table 6:** Experimental results on the Hijja and AHCD through noise injection.

	Type of noise injection with using Nadam algorithm					
	Gaussian noise		Salt-and-pepper noise		Speckle noise	
	Training accuracy	Testing accuracy	Training accuracy	Testing accuracy	Training accuracy	Testing accuracy
AHCD	99.17	96.78	99.16	97.15	99.14	96.82
Hijja	97.18	89.85	92.96	90.10	91.32	89.73

**Table 7:** Experimental results on mixing of Hijja and AHCD through different data augmentation techniques.

Mixed dataset		Data augmentation techniques with using Nadam algorithm									
		Rotation		Shifting		Rotation and shifting		Flipping		Gaussian noise	
Training	Testing	Training accuracy	Testing accuracy	Training accuracy	Testing accuracy	Training accuracy	Testing accuracy	Training accuracy	Testing accuracy	Training accuracy	Testing accuracy
80(%): AHCD 20(%): Hijja	20(%): AHCD 10(%): Hijja	99.62	97.42	99.36	98.02	99.38	**98.32**	99.77	97.08	99.15	96.74
80(%): Hijja 20(%): AHCD	20(%): Hijja 10(%): AHCD	97.36	88.47	93.66	89.07	94.91	**90.54**	95.21	88.21	96.68	88.49
80(%): AHCD 80(%): Hijja	20(%): AHCD 20(%): Hijja	97.27	74.53	97.16	75.13	98.13	**78.13**	98.16	74.22	96.98	74.02

## Data Availability

Previously reported AHCD data were used to support this study and are available at https://www.kaggle.com/mloey1/ahcd1. These prior studies (and datasets) are cited at relevant places within the text as [43].

## Abbreviations

AHCR: Arabic handwritten characters recognition
DL: Deep learning
CNNs: Convolution neural networks
AHCD: Arabic handwritten character dataset
SVM: Support vector machine
ADBase: Arabic digits database
HACDB: Handwritten Arabic characters database
OIHACDB: Offline handwritten Arabic character database
CDCGAN: Conditional deep convolutional generative adversarial network
Tanh: Hyperbolic tangent
ReLU: Rectified linear unit
CE: Cross-entropy
GD: Gradient descent
NAG: Nesterov accelerated gradient
TP: True positive
FP: False positive
FN: False Negative
TN: True negative
AUC: Area under curve
ROC: Receiver operating curve
ELU: Exponential linear unit

## Symbols

*I*: Image
*m*: Width and height of the image
*c*: Number of channels
*F*: Filter
*n*: Filter size
⊗: Convolution operation
*C*: Convolution map
*a*: Size of convolution map
*S*_*c*_: Stride
*p*: Padding
*f*: Nonlinear activation function
*C*_*a*_: Convolution map after applying *f*
*K*: Kernel
*S*_*p*_: Number of patches
pool: Pooling operation
*P*: Pooling map
*Y* ^0^: Concatenation vector
*P*_*i*_^*c*^: Output of the convolution layer
*P*_*i*−1_^*c*−1^: Convoluted image
*Y* ^*i*−1^: Input of the *i* th fully connected hidden layer
*Y* ^*i*^: Output of the *i* th fully connected hidden layer
*H* ^*i*^: Weight sum vector
*B* ^*i*^: Bias
*E*: Cost function
${\overset{ˇ}{y}}_{i}$: Desired output
*ϕ*: Update of the filter *F*
(∂*E*/∂*φ*): Gradient
*α*: Model learning
*m*: Momentum
*v*(*t*): Moment gained at the *i* th iteration
*ε*: Smoothing value
*G*(*t*): Sum of the squares of the gradient
*E*[*G*(*t*)^2^]: Decaying overage
*r*(*t*): Moments vector
*β*: Decay rate
*r*(*t* − 1): Mean of the previous gradient
*v*(*t* − 1): Variance of the previous gradient.
